# Analysis of Massive Transfusion Protocol Utilization in Trauma Across Sociodemographic Groups

**DOI:** 10.3390/medicina61071133

**Published:** 2025-06-24

**Authors:** Monique Arnold, Bharti Sharma, Matthew Conn, Kate Twelker, Navin D. Bhatia, George Agriantonis, Jasmine Dave, Juan Mestre, Zahra Shafaee, Jennifer Whittington

**Affiliations:** 1 Department of Emergency Medicine, Icahn School of Medicine at the Mount Sinai Hospital, New York, NY 10029, USA; monique.arnold@mountsinai.org; 2 Department of Surgery, NYC Health + Hospitals/Elmhurst Hospital Center, Queens, NY 11373, USA; mconn@jhmc.org (M.C.); twelkerk1@nychhc.org (K.T.); bhatian1@nychhc.org (N.D.B.); agriantg@nychhc.org (G.A.); davej@nychhc.org (J.D.); mestreju@nychhc.org (J.M.); shafaeez1@nychhc.org (Z.S.); harrisj20@nychhc.org (J.W.); 3 Department of Surgery, Icahn School of Medicine at the Mount Sinai Hospital, New York, NY 10029, USA

**Keywords:** massive transfusion, transfusion, platelets, trauma, emergency

## Abstract

*Background and Objectives*: Blood shortages are a national crisis, creating dangerous scenarios for patients requiring the use of a massive transfusion protocol (MTP). A judicious use of blood products is critical to rescue salvageable patients while refraining from unnecessary MTP to save precious resources. This study examines effect of trauma characteristics, socioeconomic variables and markers of futility on the likelihood of activating and receiving MTP in the trauma setting. *Materials and Methods*: In this retrospective study, emergency department (ED) trauma activations from a database of an urban Level I trauma center were analyzed from 1 January 2017 to 30 June 2022, inclusive. In-ED mortality, RBC transfusion volumes during initial resuscitation, patient sociodemographic data, and trauma event factors were analyzed. The primary outcomes were the dichotomous outcomes of MTP activation and MTP transfusion. Univariable analyses and logistic regressions were conducted, with class balancing sensitivities applied to the multivariable regressions to adjust for imbalance in the data. *p* < 0.05 was considered statistically significant. *Results*: Among the 8670 trauma activations, there was a 0.3% in-ED mortality rate. MTP activation and MTP transfusion were associated with higher in-ED mortality rates (3.8% and 15.4%, respectively, compared to 0.2% without MTP). Younger patients, male patients, and Medicaid recipients were more likely to undergo MTP activation; Medicare patients were less likely. Penetrating trauma substantially increased the likelihood of both MTP activation (odds ratio (OR) 5.81) and transfusion (OR 3.63). The logistic regression models identified the presence of penetrating trauma, lower probability of survival, and age as the most important covariates. Models demonstrated high discriminatory value (area under the curve (AUC) of the receiver operating characteristic curve (ROC) of 0.876 for MTP activation, 0.935 for MTP transfusion) and precision (0.974 for activation, 0.994 for transfusion), with class balancing further improving model performance and precision scores. *Conclusions*: These results are significant as assessing the futility of MTP should be equitable, and future transfusion guidelines should consider salvageability in cases with a low probability of survival despite age and mechanism.

## 1. Background

Blood shortages create challenges for healthcare facilities when treating patients and safely performing life-sustaining interventions [1,2]. Notably, there is a massive strain on blood banks in the United States, leading to shortages of vital blood components, which is especially dire for trauma patients requiring transfusions for resuscitation. Trauma patients often require the transfusion of massive amounts of blood for resuscitation, but as emphasized by recent literature, the demand for blood outpaces the supply [2]. Though the causes of the significant blood shortage in the United States are multifaceted, increased use of hemostatic resuscitation for trauma patients, whereby patents are resuscitated with large quantities of blood products and whole blood, has contributed immensely to this shortage [2].

Historically, massive transfusion protocol (MTP) has often referred to the transfusion of more than 10 units of blood products in a 24 h period [3,4]. This definition does not address the need for blood in a shorter time span, as is often relevant for ED trauma patients. In the recent literature, MTP in the ED has often referred to at least five units of blood products in fixed ratios (usually 1:1:1 packed red blood cells (pRBC)/fresh frozen plasma (FFP)/platelets) transfused within an hour for the management of hemorrhagic shock [4,5,6,7]. Although these are the definitions used at many centers including ours, the optimal ratio of blood products remains contested and there is no standard definition for the minimum number of units and timeframe that constitutes MTP [4,5,6]. This limits the generalizability of studies referencing or investigating the use of MTP. Nevertheless, the utilization of MTP dramatically affects blood inventory and availability—although only up to 5% of traumas require massive transfusions, they use up to 70% of all blood transfused in trauma centers [1,8,9,10,11].

Given this mismatch in demand and supply for blood, there exists a need to triage who receives blood products and how much they receive; however, there are currently no clear guidelines for this triage. The decision to activate MTP is at the institution and the physicians’ discretion during a trauma resuscitation, but is generally triggered when a patient requires a significant amount of blood products in a short timeframe [1,10,11]. Despite the development of markers of injury severity such as injury severity score (ISS), probability of survival (Ps), and scoring systems for bleeding in trauma, such as the assessment of blood consumption (ABC) score, shock index, traumatic bleeding severity score (TBSS), and trauma-associated severe hemorrhage (TASH) score, there are currently no clear, universally accepted criteria for who will most benefit from MTP or when to activate MTP in severely injured trauma patients [12,13,14,15,16,17]. Moreover, patient mortality despite receiving MTP may indicate the futility of transfusion, which should not be tolerated when resources are scarce. Several factors may contribute to mortality in patients receiving MTP, including age, mechanism of trauma, initial Glasgow Coma Scale Score (GCS), injury severity, comorbidities, and the total amount of blood transfused at 4 h and 24 h [12].

There are some promising recent efforts for predicting futility, with some recent work focusing on developing clinical decision support tools to identify futility earlier in trauma resuscitation in the ED and pre-hospital settings [18,19,20,21,22,23]. For example, the Futility of Resuscitation Measure (FoRM) score, a model for predicting the futility of resuscitation among older adult trauma patients, finds that the most important predictors of futility of resuscitation are massive transfusion of 16 units of pRBC or more within 4 h, prehospital cardiac arrest, severe traumatic brain injury (TBI), profound hypotension, and performance of ED thoracotomy [18]. The Suspension of Transfusions and Other Procedures (STOP) criteria for predicting futility in severely injured trauma patients arriving in extremis finds that when field and ED physiological values are combined, severely elevated lactate and high fibrinolysis were 100% predictive of mortality, thereby establishing cut points to guide early decisions for discontinuing aggressive treatment [20]. The utilization of such tools at the bedside during trauma resuscitation has tremendous implications for improving resource allocation, anchoring life-saving resuscitative efforts in principles of beneficence, non-maleficence, and futility. As of yet, however, there no widely used or universally agreed upon metrics for predicting futility.

Even as evidence-based tools such using clinical parameters show the potential to enhance decision-making regarding the allocation of scarce resuscitative resources, emerging data underscore the significant influence of sociodemographic and structural factors on trauma outcomes. For example, race, ethnicity, and insurance status independently predict outcome disparities after trauma—specifically, data from a study of almost 430,000 trauma patients shows that African American and Hispanic patients are more likely to be uninsured and to sustain penetrating trauma than White patients, and that African American patients, Hispanic patients, and uninsured patients have worse outcomes during traumas [24]. Insurance status had the strongest association with mortality after trauma, with results showing that uninsured patients were almost 50% more likely to die as a result of trauma [24].

These disparities reflect broader inequities within the United States’ mixed healthcare insurance system, which combines government-funded programs with employer-sponsored private insurance, individual market plans, and a significant uninsured population, creating fragmented coverage that often falls along racial, ethnic, geographic, and socioeconomic lines [25,26]. The government-funded programs include Medicare, a federal health insurance program primarily for Americans aged 65 and older and those with certain disabilities, and Medicaid, a joint federal–state program providing health coverage for low-income individuals and families, with eligibility varying by state [27].

This paper examines whether insurance status affects the likelihood of activating and receiving MTP, and how this interacts with other socioeconomic variables and markers of futility. Assessing the futility of MTP is important to better understand and improve future transfusion guidelines, with these disparities in mind. To date, this is the first large study focusing on the association of these factors with MTP utilization and mortality in trauma regardless of blood supply. Our study supports the argument that assessing the futility of MTP should be equitable, and future transfusion guidelines should consider salvageability in cases with a low probability of survival despite age and mechanism.

## 2. Methods

### 2.1. Data Collection

After obtaining Institutional Review Board approval with a waiver of informed consent (Elmhurst Hospital Center, Queens, New York, NY, USA), we conducted a retrospective cohort study of all ED trauma activations at Elmhurst Hospital, an urban Level I Trauma Center in Queens, New York from January 2017 to June 2022, inclusive. Patients 18 years of age and older who arrived due to trauma activation were included. Trauma activation is defined at our institution as the alerting and summoning of the trauma team of emergency department physicians and nurses and trauma surgery physicians to the trauma bay when a trauma patient is deemed to have a moderate or severe risk of injury based on the pre-established trauma guidelines at our trauma center. During trauma activations, laboratory, blood bank, and radiologic technologist staff are also notified and placed on standby to provide appropriate emergent services. Patients who arrived dead (no discernible blood pressure, pulseless, apneic) were excluded.

MTP activations refer to patients for whom MTP was activated (flagged in the data), while MTP transfusions refer specifically to patients who received a significant number of pRBCs during their treatment in the ED. These patients were compared to patients who did not have MTP activated or transfused in the ED. MTP was defined as greater than 5 units of blood product within the first 6 h during their ED stay, consistent with the literature [1]. RBC transfusion volume during initial resuscitation, as well as patient demographics, trauma event characteristics, payer status, and standardized markers of futility including ISS and Ps were recorded. Records with missing data for age, sex, race, ethnicity, or payer status were excluded from the data.

### 2.2. Univariable Analyses

Generally, between-group medians were compared using the non-parametric Wilcoxon rank sum test for comparing the median values of two groups and the non-parametric Kruskal–Wallis rank sum test for comparing the median values of three or more groups. Pearson’s correlation coefficient was employed to assess the linear relationship between the ISS and Ps variables.

Univariable regression analyses of a priori-determined variables (age, sex, race, ethnicity, Medicaid or Medicare insurance status, trauma type, ISS, and Ps) were conducted. For numeric variables, we used the Wilcoxon rank sum test or the Kruskal–Wallis rank sum test for comparing medians, as previously described. Categorical variables were checked with the χ^2^ test or Fisher’s exact test if the number of observations was <20. Odds ratios (OR), standard errors (SE), confidence intervals (CI), and *p*-values for the association of each variable with MTP activation and MTP transfusion were determined.

### 2.3. Multivariable Analyses

Multivariable logistic regression models were constructed to analyze the relationship between the sociodemographic and trauma variables with MTP activation and MTP transfusion. ORs, 95% CIs, and *p*-values for each covariate in the regression models were determined. MTP activation and transfusion were rare events in the dataset, leading to class imbalances in the data. To avoid biases due to class imbalances, we performed additional regression sensitivities in which we tested six class balancing techniques as outlined below [28,29,30,31].

1. *Weighting using inverse class frequencies*—balancing by scaling the weight of each class to the inverse of its frequency, thereby assigning higher weights to the minority class and lower weights to the majority class.
2. *Weighting using means*—balancing by scaling the weight of each class to the inverse of its frequency, similarly assigning higher weights to the minority class and lower weights to the majority class.
3. *Downsampling* (undersampling)—randomly subsetting (removing or reducing) the majority classes in the training set so that their class frequency matches the minority class.
4. *Upsampling* (oversampling)—randomly subsetting (and replacing with artificial or duplicate data points) the minority classes in the training set so that their class frequency matches the majority class.
5. *Synthetic minority oversampling technique (SMOTE)*—a hybrid method that downsamples the majority class and synthesizes new data of the minority class using the k-nearest neighbor algorithm.
6. *Random oversampling examples (ROSE)*—a hybrid method that utilizes majority downsampling and minority upsampling to synthesize new data from both classes.

### 2.4. Performance Evaluation

The assessments of the models include the area under the curve (AUC) of the receiver operating characteristic curve (ROC) and precision scores (area under the precision–recall curves (AUPRC). AUC was calculated to assess the discriminatory power of the original models and the model sensitivities. AUC is a proxy for accuracy in predicting a binary outcome, with AUCs closer to 1 being more predictive. The 95% CI for the AUC for each model was also noted. Precision scores were calculated to measure the overall precision of the models in predicting positive cases. Precision scores quantify the proportion of accurately predicted positive instances among all instances predicted as positive.

All data were analyzed using R version 4.3.1 (“Beagle Scouts” version, released 6 June 2016) and RStudio Version 2023.09.1+494. Statistical significance was defined as *p* < 0.05 for significance at the 95% CI, *p* < 0.01 at the 99% CI, and *p* < 0.001 at the 99.99% CI.

## 3. Results

There were 8670 trauma activations. Table 1 shows the socio-demographic and trauma characteristics of the patient sample segregated by MTP activation and MTP transfusion. A total of 90% were traumas due to blunt force, and 10% were due to penetrating trauma. A total of 66% of the sample was male, with a mean age of 53.7 years; the majority of the trauma patients were non-Hispanic (64%) and their race was identified as “Other” (56%). The majority of patients were on Medicaid (33%) or Medicare (27%). Only 3% (*n* = 265) of traumas saw an MTP activation; of those, there were 213 traumas for which MTP was activated but <5 units of pRBCs were given. As such, only 0.5% of traumas saw an actual MTP transfusion. The median amount of pRBC for those with MTP activations was two units and for those with MTP transfusions was five units, a statistically significant difference (*p* < 0.01).

The univariable analysis in Table 2 shows that younger patients were more likely to have MTP activated (*p* < 0.001) and transfused (*p* < 0.05). Interestingly, men were almost twice as likely to have MTP activated (OR 1.83, 95% CI 1.37–2.48, *p *< 0.001), but there was no statistically significant difference for sex for MTP transfusion (OR 1.52, 95% CI 0.83–2.96, *p* = 0.18). Though non-Hispanic patients were slightly less likely to have MTP activated (OR 0.76, 95% CI 0.60–0.98, *p* < 0.05), there was no statistically significant difference for MTP transfusion (OR 0.84, 95% CI 0.49–1.49, *p* = 0.55). Likewise, there was no statistically significant difference in MTP activation or MTP transfusion based on race (*p >* 0.05). Patients on Medicare were much less likely to have MTP activated (OR 0.32, 95% CI 0.22–0.47, *p* < 0.001) or have MTP transfused (OR 0.22, 95% CI 0.07–0.55, *p *< 0.001). Patients on Medicaid were more likely to have MTP activated (OR 1.30, 95% CI 1.00–1.66, *p *< 0.05), but there was no statistically significant difference in MTP transfusion (OR 1.42, 95% CI 0.80–2.46, *p* = 0.22).

MTP activation and transfusion also varied with trauma characteristics. Specifically, patients with penetrating trauma were almost six times more likely to have MTP activated (OR = 5.81, 95% CI 4.47–7.52, *p* < 0.001) and almost four times more likely to have MTP transfused (OR 3.63, 95% CI 1.93–6.50, *p* < 0.001). Trauma type had the largest effects (largest ORs) of all the variables tested. ISS was significantly higher in patients with MTP activations (*p *< 0.001) and MTP transfusions (*p *< 0.001). The median Ps was significantly lower in MTP activations (*p *< 0.001) and MTP transfusions (*p *< 0.001). ISS and Ps were negatively correlated (tau = −0.520, *p* < 0.001).

The overall in-ED mortality rate was 0.3%. As expected, in-ED mortality was higher with higher ISS and with lower Ps—the median ISS for those who died in the ED was 30 versus 4 for those who did not, a statistically significant difference (*p* < 0.001); the median Ps for those who died in the ED was 0.191 versus 0.983 for those who did not, also a statistically significant difference (*p* < 0.001). Table 3 shows comparison of in-ED mortality with MTP. MTP activation and MTP transfusion were associated with in-ED mortality—those with MTP activations had a higher rate of in-ED mortality than those without (3.8% vs. 0.2%, *p* < 0.001). Similarly, those receiving MTP transfusions had a higher rate of in-ED mortality than those without (15.4% vs. 0.2%, *p* < 0.001). The discriminatory value of MTP activation on in-ED mortality was high at 0.703 (95% CI 0.599–0.806), while the discriminatory value of MTP transfusion was slightly lower at 0.671 (95% CI 0.572–0.771).

The original multivariable logistic regression models were a priori constructed to include age, sex, race, ethnicity, Medicaid or Medicare status, trauma type, ISS, and Ps, as well as an interaction variable between ISS and Ps (ISS*Ps), given their high degree of correlation. The results are shown in Table 4. Once again, penetrating trauma had the largest effect, with those with penetrating trauma being almost 10 times more likely to have MTP activated (OR 9.79, 95% CI 6.86–14.0, *p* < 0.001), and almost 4 times more likely to have MTP transfused (OR 3.92, 95% CI 1.79–8.36). Age was a significant covariate for MTP activation (OR 0.99, 95% CI 0.98–1.00, *p* < 0.05); however, it was not for MTP transfusion (*p* = 0.94). The AUC for both models was high—0.876 (95% CI 0.850–0.902) for the MTP activation model and 0.935 (95% CI 0.895–0.974) for the MTP transfusion model. Precision scores were also high—0.974 for MTP activation and 0.994 for MTP transfusion.

Table 5 shows the AUCs, 95% CI, and precision scores for the original models and each class balancing sensitivity. All models except the weighting using frequency model had higher AUCs and precision scores than the original regression. AUCs ranged from 0.875 to 0.881 for MTP activation and 0.933 to 0.946 for MTP transfusion across the models. Precision ranged from 0.970 to 0.992 for MTP activation and 0.994 to 1.000 for MTP transfusion across the different models.

## 4. Discussion

This is the first study focusing on massive transfusion utilization variation with insurance status, and how this interacts with other socioeconomic variables and markers of futility. Overall, of the 8670 trauma activations, only 3.1% saw MTP activations and 0.5% saw MTP transfusions. There was high in-ED mortality, higher ISS, and lower Ps for those for whom MTP was activated. Patients on Medicare were less likely to have MTP activated, while patients on Medicaid were more likely to have MTP activated. Additionally, men, younger patients, and patients with penetrating trauma were more likely to have MTP activated; no significant difference in MTP activation was found concerning race. The most important factors associated with MTP activation were penetrating trauma and Ps.

Univariable analyses indicated that patients with a younger age, male gender, and receiving Medicaid were more likely to undergo MTP activation, while those on Medicare were less likely. Penetrating trauma significantly increased the likelihood of both MTP activation (OR 5.81) and transfusion (OR 3.63). Multivariable logistic regression models, including age, sex, race, ethnicity, Medicaid or Medicare status, trauma type, ISS, and Ps, confirmed the substantial impact of penetrating trauma, making individuals almost 10 times more likely to have MTP activated and four times more likely to have MTP transfused. Models demonstrated high discriminatory values (AUC 0.876 for activation, 0.935 for transfusion) and precision scores (0.974 for activation, 0.994 for transfusion). Class balancing sensitivity improved model performance across various models.

The sociodemographic profile of trauma patients in our sample differed substantially from that of the surrounding District 3 (Jackson Heights and East Elmhurst), as per the 2023 U.S. Census ACS five-year estimates [32]. Our cohort was older (median age 53 vs. 39.5 years), predominantly male, and majority non-Hispanic (64%), in contrast to the district’s largely Hispanic population (61.9%) and balanced gender distribution [32]. Insurance coverage also differed, with Medicaid (33%) and Medicare (27%) being most common in our sample, whereas district residents more often reported employer-based plans (30.5%). Notably, Elmhurst Hospital draws patients from a broad catchment area beyond District 3, which may help explain the sociodemographic differences observed between our trauma sample and the district-level population data [32].

### 4.1. Age, Transfusion, and Mortality

Prior research shows that older patients are more likely to undergo RBC transfusions and MTP activation than younger patients [12,13,33,34]. Our data, however, refutes this, as younger patients were more likely to have MTP activated or transfused. Lim et al. (2018) showed that 32 out of 58 patients with hemorrhagic shock sustained from trauma received MT, with a mean age 11 years younger than those who did not, a statistically significant difference [35]. This large age difference may be explained by the fact that age is a predictor of mortality after trauma, with mortality increasing with age in massively transfused patients [36]. Another retrospective study showed that the association between plasma-to-RBC ratio and in-hospital mortality in non-geriatric patients was clearly shown; however, the relationship between plasma-to-RBC ratio with mortality among geriatric patients remained inconclusive in a trauma setting [37]. Based on these studies, there seems to be a statistically significant difference among ages in the outcome of MTP given in a traumatic setting.

### 4.2. Sex, Transfusion, and Mortality

Numerous studies have shown that most patients receiving MTP are male; our results are consistent with this finding [38,39]. In the secondary analysis of the Pragmatic Randomized Optimal Platelet and Plasma Ratios (PROPPR) trial, female patients were shown to receive fewer units of total blood products than their male counterparts after hemostasis was achieved [40]. Specifically, women received lower volumes of all products, with a 38% reduction in FFP, 49% reduction in platelets, and 49% reduction in the volume of RBCs; however, there was no difference in transfusion requirement during active hemorrhage [40]. Finally, men have been shown to have improved outcomes after massive transfusion as compared to women. For example, in a retrospective study of 704 MTP-receiving patients at 23 Level I trauma centers, male patients receiving a high plasma-to-RBC ratio had lower 24 h- and 30-day mortality rates, while women had no improvements in mortality rates [41].

### 4.3. Age, Ethnicity, Race, and Insurance Status

Our data show that Medicaid patients were more likely to have MTP activated or transfused, while Medicare patients were less likely. Age, ethnicity, and race are intricately correlated with insurance status in the US. In 2010, when the Affordable Care Act (ACA) was passed, seventy percent of non-Hispanic Black non-elderly adults were more likely to be uninsured than non-Hispanic White adult patients [42]. Such disparities in insurance coverage result in poorer access to care leading to worse health outcomes [42,43,44,45,46]. As the number of Black and Hispanic beneficiaries has grown over time, Medicaid has played an increasingly vital role as a source of coverage for racial minorities [47]. Additionally, age is clearly correlated with Medicare access, as, as of 2021, the majority of Medicare beneficiaries (86%) are aged 65 and older, with the remaining 14% qualifying for Medicare because of a long-term disability [48].

### 4.4. Clinical Significance

These data underscore important trends in transfusion practice and raise critical questions about the use of MTP in patients with severe injuries. The decision to transfuse MTP must be judicious and restrictive, and MTP guidelines remain largely subjective. Patients who received MTP had a higher ISS and lower Ps, indicating lower chances of survivability and possibly higher risks of futility of transfusion. Yet, it can be argued that for these patients, from the trauma providers’ perspectives, without MTP, fatality is inevitable. This poses the question of how to balance the distribution of blood for those with urgent needs with those who are likely to most benefit when these do not substantially overlap.

Our models demonstrated high AUC and precision for predicting both MTP activation and transfusion. These predictive performances suggest that, with further external validation, such models could eventually assist clinicians in making earlier, more data-informed decisions during trauma resuscitation. In doing so, they hold promise for improving patient outcomes while preserving finite resources.

### 4.5. Strengths

An important strength of this study is that, as previously discussed, it delineates MTP activation from MTP transfusion. The decision to activate MTP, as noted, is complex and discretionary. MTP transfusion is impacted by factors including, but not limited to, patient expiration before transfusion is complete, rapidly reversible hemorrhage, or early termination of ongoing MTP due to perceived futility by the clinical team. By explicitly distinguishing between activation and actual transfusion, this study provides a more nuanced understanding of how MTP is utilized in real-world trauma settings, which has been underexplored in the prior literature.

Though patients for whom MTP was activated and transfused were a small proportion of the population, our use of class balancing techniques for adjusting for class imbalances reduces bias towards the majority class. These techniques are very popular in the scientific literature; for example, SMOTE is one of the most popular preprocessing techniques, and is considered one of the standards in the framework of balancing imbalanced data [28,29,30,31]. This methodological rigor strengthens the validity of our predictive models, despite the rarity of some outcomes.

## 5. Limitations

The limitations of this study include the retrospective design, which is associated with a high degree of missing or incomplete documentation. As is common with electronic health record data, this limitation reduced our ability to incorporate all potentially relevant variables into the analysis. Additionally, as a single-center study, the findings may not be generalizable and should be validated in other trauma centers.

Additionally, as noted, a large proportion of patients for whom MTP was activated ultimately received fewer than five units of pRBCs in the ED. Potential reasons for this, as highlighted above, include early mortality, rapidly reversible hemorrhage, and early termination. Given the high rate of ED mortality among MTP activation cases, early death or early abortion of the MTP process are plausible explanations. However, detailed chart review to confirm the exact rationale in each case was beyond the scope of this study. This limitation may affect the interpretability and generalizability of our findings, particularly in trauma systems with different MTP activation criteria or transfusion practices.

An additional limitation is our lack of direct incorporation of clinical factors affecting MTP activation. For example, vital signs, GCS on arrival, and prior resuscitation efforts have been shown to affect MTP activation in prior studies but were not directly assessed in our study. These factors, however, are incorporated in the calculations for ISS and Ps, and so their effects are indirectly measured. Other established early predictive tools for massive transfusion (such as the aforementioned ABC score, shock index, TBSS and TASH score) or newer tools (such as the aforementioned FoRM score and STOP criteria) were also not included in our model. Though these scores are not frequently used at bedside in our center, their omission limits the applicability of our findings to settings where such scores are routinely used for decision-making. Future research should evaluate how integrating these dynamic, point-of-care tools could enhance the performance and generalizability of predictive models for MTP activation and futility.

Finally, while our study examined overall mortality, we did not evaluate differential outcomes such as mortality across sociodemographic subgroups. Such disparities have been extensively documented in the existing literature and were outside the scope of the present investigation.

## 6. Conclusions

In conclusion, our institutional data demonstrate that though insurance status has some effect on MTP activation, penetrating trauma and probability of survival are the most important factors associated with MTP activation. These findings are important as assessing the futility of MTP should be equitable, and future transfusion guidelines should consider salvageability, even in cases with a low probability of survival regardless of age and mechanism. Resource stewardship in trauma care is a critical issue given the scarcity and cost of blood products. By examining patterns of transfusion and their associations with outcomes, the study contributes valuable insights into when MTP may or may not confer benefits, and where opportunities may exist to improve decision-making and avoid futile interventions.

## Figures and Tables

**Table 1:** Patient socio-demographic and trauma characteristics segregated by MTP activation and MTP transfusion.

		MTP Activated		MTP Transfused	
Characteristic	Overall, N = 8670 ^1^	NO, N = 8405 ^1^	YES, N = 265 ^1^	NO, N = 8618 ^1^	YES, N = 52 ^1^
**AGE**	53.05 (24.23)	53.64 (24.30)	38.60 (19.43)	53.11 (24.26)	43.81 (17.22)
**SEX**					
Female	2909 (34%)	2851 (34%)	58 (22%)	2896 (34%)	13 (25%)
Male	5761 (66%)	5554 (66%)	207 (78%)	5722 (66%)	39 (75%)
**RACE**					
American Indian	5 (<0.1%)	5 (<0.1%)	0 (0%)	5 (<0.1%)	0 (0%)
Asian	1138 (13%)	1109 (13%)	29 (11%)	1131 (13%)	7 (13%)
Black	809 (9.3%)	780 (9.3%)	29 (11%)	805 (9.3%)	4 (7.7%)
Native Hawaiian or Another Pacific Islander	7 (<0.1%)	6 (<0.1%)	1 (0.4%)	7 (<0.1%)	0 (0%)
Other ^2^	4843 (56%)	4681 (56%)	162 (61%)	4811 (56%)	32 (62%)
White	1868 (22%)	1824 (22%)	44 (17%)	1859 (22%)	9 (17%)
**ETHNICITY**					
Hispanic Origin	3154 (36%)	3041 (36%)	113 (43%)	3133 (36%)	21 (40%)
Non-Hispanic Origin	5516 (64%)	5364 (64%)	152 (57%)	5485 (64%)	31 (60%)
**INSURANCE STATUS**					
Medicaid	2818 (33%)	2717 (32%)	101 (38%)	2797 (32%)	21 (40%)
Medicare	2338 (27%)	2309 (27%)	29 (11%)	2334 (27%)	4 (7.7%)
No Charge	889 (10%)	847 (10%)	42 (16%)	879 (10%)	10 (19%)
Other	1059 (12%)	1022 (12%)	37 (14%)	1054 (12%)	5 (9.6%)
Private	1566 (18%)	1510 (18%)	56 (21%)	1554 (18%)	12 (23%)
**TRAUMA TYPE**					
Blunt	7789 (90%)	7623 (91%)	166 (63%)	7752 (90%)	37 (71%)
Penetrating	881 (10%)	782 (9.3%)	99 (37%)	866 (10%)	15 (29%)
**PROBABILITY OF SURVIVAL**	0.98 (0.10)	0.98 (0.08)	0.96 (0.29)	0.98 (0.09)	0.70 (0.33)
**ISS**	4.00 (6.92)	4.00 (6.27)	17.00 (13.25)	4.00 (6.71)	25.50 (13.23)
**PRBCs transfused**	0.00 (0.67)	0.00 (0.16)	2.00 (2.66)	0.00 (0.38)	5.00 (2.93)
**IN-ED MORTALITY**					
NO	8647 (100%)	8392 (100%)	255 (96%)	8603 (100%)	44 (85%)
YES	23 (0.3%)	13 (0.2%)	10 (3.8%)	15 (0.2%)	8 (15%)

^1^ Median (SD); n (%) ^2^ “Other” includes all other responses not included in the “White”, “Black”, “American Indian”, “Asian”, and “Native Hawaiian or Another Pacific Islander” race categories described above, including any write-in responses.

**Table 2:** Univariable analysis of patient socio-demographic and trauma characteristics as predictors of MTP activation and MTP transfusion in trauma patients.

	MTP Activation				MTP Transfusion			
Characteristic	OR ^1^	SE ^1^	95% CI ^1^	*p*-Value ^2^	OR ^1^	SE ^1^	95% CI ^1^	*p*-Value ^2^
**Age**	0.98	0.003	0.98, 0.99	<0.001 ***	0.99	0.006	0.98, 1.00	0.042 *
**Race = White**				0.14				0.96
American Indian	0	239			0	1073		
Asian	1.08	0.242	0.67, 1.73		1.28	0.505	0.46, 3.44	
Black	1.54	0.243	0.95, 2.47		1.03	0.602	0.28, 3.16	
Native Hawaiian or Other Pacific Islander	6.91	1.09	0.36, 41.6		0	907		
Other	1.43	0.172	1.03, 2.03		1.37	0.378	0.68, 3.06	
**Sex = Male**	1.83	0.15	1.37, 2.48	<0.001 ***	1.52	0.321	0.83, 2.96	0.18
**Ethnicity = Non-Hispanic Origin**	0.76	0.126	0.60, 0.98	0.033 *	0.84	0.284	0.49, 1.49	0.55
**Mechanism = Penetrating Trauma**	5.81	0.132	4.47, 7.52	<0.001 ***	3.63	0.308	1.93, 6.50	<0.001 ***
**ISS**	1.13	0.006	1.12, 1.15	<0.001 ***	1.12	0.01	1.10, 1.15	<0.001 ***
**Ps**	0.01	0.256	0.01, 0.02	<0.001 ***	0.01	0.364	0.00, 0.02	<0.001 ***
**Medicare**	0.32	0.198	0.22, 0.47	<0.001 ***	0.22	0.521	0.07, 0.55	<0.001 ***
**Medicaid**	1.3	0.129	1.00, 1.66	0.046 *	1.42	0.284	0.80, 2.46	0.22

^1^ OR = odds ratio, SE = standard error, CI = confidence interval. ^2^ * *p* < 0.05; ** *p* < 0.01; *** *p* < 0.001.

**Table 3:** Comparison of in-ED mortality with MTP activation and MTP transfusion.

	In-ED Mortality		
	NO	YES	*p*-Value ^1,2^
**MTP Activation**			<0.001 ***
NO	8392 (99.8%)	13 (0.2%)	
YES	255 (96.2%)	10 (3.8%)	
**Total**	8647 (99.7%)	23 (0.3%)	
**MTP Transfusion**			<0.001 ***
NO	8603 (99.8%)	15 (0.2%)	
YES	44 (84.6%)	8 (15.4%)	
**Total**	8647 (99.7%)	23 (0.3%)	

^1^ Fisher’s exact test. ^2^ * *p* < 0.05; ** *p* < 0.01; *** *p* < 0.001.

**Table 4:** Multivariable analysis of predictors of MTP activation and MTP transfusion in trauma patients.

	MTP Activation					MTP Transfusion		
Characteristic	OR ^1^	SE ^1^	95% CI ^1^	*p*-Value ^2^	OR ^1^	SE ^1^	95% CI ^1^	*p*-Value ^2^
**(Intercept)**	2.72	0.75	0.62, 11.8	0.18	0.26	1.16	0.03, 2.48	0.24
**Age**	0.99	0.004	0.98, 1.00	0.006 **	1	0.009	0.98, 1.02	0.94
**Race = White**								
American Indian	0	387		0.98	0	1048		>0.99
Asian	0.84	0.269	0.49, 1.41	0.51	0.82	0.537	0.27, 2.34	0.71
Black	0.89	0.284	0.50, 1.54	0.68	0.68	0.654	0.17, 2.31	0.55
Native Hawaiian or Other Pacific Islander	2.51	1.39	0.09, 25.2	0.51	0	788		0.99
Other	0.76	0.233	0.48, 1.20	0.23	0.81	0.488	0.31, 2.15	0.66
**Gender = Male**	0.84	0.179	0.60, 1.20	0.34	0.8	0.365	0.40, 1.69	0.55
**Ethnicity = Non-Hispanic** **Origin**	0.87	0.189	0.60, 1.25	0.45	1.14	0.4	0.51, 2.47	0.74
**Medicaid**	0.9	0.156	0.66, 1.22	0.5	1.38	0.326	0.72, 2.61	0.33
**Medicare**	0.69	0.266	0.41, 1.16	0.17	0.37	0.621	0.10, 1.18	0.11
**Mechanism = Penetrating**	9.79	0.183	6.86, 14.0	<0.001 ***	3.92	0.391	1.79, 8.36	<0.001 ***
**ISS**	0.98	0.017	0.95, 1.01	0.27	0.99	0.02	0.95, 1.03	0.69
**PS**	0	0.646	0.00, 0.01	<0.001 ***	0	0.871	0.00, 0.01	<0.001 ***
**ISS × PS**	1.19	0.019	1.15, 1.24	<0.001 ***	1.19	0.028	1.13, 1.26	<0.001 ***

^1^ OR = odds ratio, SE = standard error, CI = confidence interval. ^2^ * *p* < 0.05; ** *p* < 0.01; *** *p* < 0.001.

**Table 5:** Performance metrics of logistic regression models.

	MTP Activation		MTP Transfusion	
Model Sensitivity	AUC (95% CI)	Precision	AUC (95% CI)	Precision
**Original**	0.876 (0.850–0.902)	0.974	0.935 (0.895–0.974)	0.994
**Weighting Using Frequency**	0.875 (0.848–0.901)	0.970	0.933 (0.893–0.973)	0.994
**Weighting Using Means**	0.881 (0.856–0.905)	0.992	0.946 (0.919–0.972)	0.999
**Downsampling**	0.876 (0.850–0.902)	0.992	0.939 (0.914–0.964)	1.000
**Upsampling**	0.880 (0.856–0.905)	0.992	0.945 (0.918–0.972)	0.999
**SMOTE**	0.881 (0.856–0.905)	0.992	0.945 (0.918–0.972)	0.999
**ROSE**	0.876 (0.852–0.901)	0.992	0.944 (0.914–0.974)	0.999

## Data Availability

The data presented in this study are available on request from the corresponding author due to privacy restrictions.
